# A double‐blind, randomized control trial to investigate the therapeutic potential of garlic scapes for high apoprotein E levels in a high‐Fat diet‐induced hypercholesteremic rat model

**DOI:** 10.1002/fsn3.4331

**Published:** 2024-07-31

**Authors:** Nizwa Itrat, Mahr un Nisa, Fahad Al‐Asmari, Mohamed Fawzy Ramadan, Eliasse Zongo

**Affiliations:** ^1^ Department of Nutritional Sciences, Faculty of Medical Sciences Government College University Faisalabad Punjab Pakistan; ^2^ Department of Food and Nutrition Sciences, College of Agricultural and Food Sciences King Faisal University Al‐Ahsa Saudi Arabia; ^3^ Department of Clinical Nutrition, Faculty of Applied Medical Sciences Umm Al‐Qura University Makkah Saudi Arabia; ^4^ Laboratoire de Recherche et d'Enseignement en Santé et Biotechnologies Animales Université Nazi BONI Bobo Dioulasso 01 Burkina Faso

**Keywords:** antioxidants, apoprotein E, hyperlipidemia, natural products, phytochemicals

## Abstract

Hypercholesteremia is the main contributor to metabolic diseases, including obesity, hypertension, and diabetes, which are the primary global sources of morbidity and death rates. Garlic scapes, a member of the *Allium sativum* family and a rich source of antioxidants, are utilized in various cuisine preparations due to their unique flavors and tastes. The current study examined garlic scape powder's effect on apoprotein E and its ability to decrease cholesterol. In an in vivo experiment, normal, healthy Wistar albino rats (weeks) were divided into a negative control group (NC, *n* = 10) and a high‐fat diet‐raised group (*n* = 50) until they achieved cholesterol ≥250 mg/dL. Hypercholesteremic rats were further divided randomly into five groups: positive control (PC), standard group (fenofibrate 20 mg/kg bwt), and treatment groups G1, G2, and G3 that were administered with garlic scape powder 400 mg, 800 mg, and 1200 mg/kg bwt orally, respectively, for 3 months. The blood samples were examined for cholesterol, triglycerides, high‐density lipoproteins (HDLs), low‐density lipoproteins, apoprotein E, albumin levels, alanine aminotransferase (ALT), and aspartate aminotransferase (AST). The liver tissues of the rats were subjected to histopathology. The lipid profile was assessed using serum kit techniques, whereas an ELISA kit was used to evaluate apoprotein E, and a serum kit was used to estimate ALT and AST. In comparison to all other groups except NC, the highest dose of 1200 mg/kg bwt of garlic scapes significantly (*p* ≤ .05) increased serum insulin (13.66 ± 0.72 μU/mL), apoprotein E levels (6.08 ± 0.10 mg/dL), HDL (42.1 ± 1.81 mg/dL), and reduce TG (88.7 ± 1.64 mg/dL) and decreased overall cholesterol levels (67.9 ± 1.17 mg/dL). Except for NC, all treatment groups had significantly (*p* ≤ .05) lower ALT and AST values than PC. To sum up, powdered garlic scapes may be a great way to avoid hyperlipidemia, which raises the risk of cardiovascular illnesses. ALT and AST levels were significantly (*p* ≤ .05) reduced in all treatment groups compared to PC, except for NC. In conclusion, garlic scape powder may be an excellent source to prevent hyperlipidemia, a risk factor for cardiovascular diseases. In addition, powdered garlic scapes supplementation at high doses may be used as an alternative natural source in functional foods to halt hyperlipidemia without liver toxicity in the long term.

## Graphical abstract

In the current long‐term study, rats were administered garlic scape powder at various doses for 12 weeks. The highest dose of garlic scape powder, 1200 mg/kg bwt, provided lipid lowering benefits due to increased cholesterol consumption by increasing apoprotein E levels and a better safety profile in terms of hepatotoxicity. Consumption of a higher dose of garlic scape powder without any toxic effect increases insulin sensitivity, making it an ideal functional food for consumers and diabetic patients in the long run.

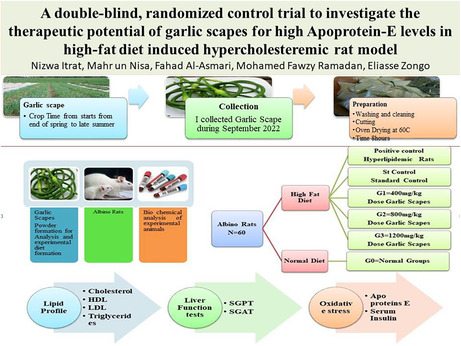

## 1 INTRODUCTION

Hyperlipidemia, often known as hypercholesterolemia, is a prevalent medical condition worldwide. The characteristic features of this phenomenon involve the presence of lipids, such as triglycerides (TG) and cholesterol, which are crucial constituents of cellular structures and perform vital functions in a range of physiological processes. Nevertheless, an abundance of lipids in the bloodstream might significantly elevate the susceptibility to cardiovascular ailments, such as myocardial infarctions, the accumulation of plaques in the arterial walls known as atherosclerosis, and cerebrovascular accidents (Irfan et al., 2019).

The prevalence and intricacy of metabolic disorders are on the rise. Hyperlipidemia is a prevalent health condition impacting around 20%–30% of the population. Women who have menstruation have an increased susceptibility to hyperlipidemia as a result of elevated amounts of the hormone estrogen. Numerous studies have demonstrated that hyperlipidemia is the primary etiological factor contributing to significant rates of morbidity and death on a global scale (Dasgupta et al., 2021).

Hyperlipidemia is a pathological disease characterized by elevated levels of lipids in the bloodstream, commonly observed in conjunction with metabolic disorders like type 2 diabetes, obesity, and hypertension. The phenomenon results in an elevation in triacylglycerol levels within the circulatory system, which progressively deteriorates. Inadequate food habits and a sedentary way of life predominantly influence hyperlipidemia. However, altering some aspects of lifestyle may be controlled (Zhang et al., 2019).

Functional and nutraceutical diets offer many advantages, such as reduced healthcare needs and expenditures. Plant‐based foods exhibit notable characteristics and are considered to be more secure in comparison to manufactured meals. This substance is widely utilized, and much study has been done to support its medical advantages (Afzaal et al., 2021). Garlic (*Allium sativum*) is distributed worldwide and is used as a food, spice, and popular remedy (Young et al., 2001). Fresh garlic scapes are seasonal vegetables, so quality after harvesting may be compromised because of varied humidity and temperature throughout the year. The nutritional content may deteriorate in real time if a cold chain system is implemented.

Garlic scapes, or *Allium sativum* var. *ophioscorodon*, are versatile and flavorful vegetables used in cooking and herbal remedies. Garlic scapes are known to contain several phytochemicals and are valued for their low cost and lack of side effects. The presence of various bioactive compounds in the plant is significant as they can help with multiple health conditions, such as hyperglycemia, the risk of cardiovascular diseases, and the effects of lipids on arterial cholesterol levels (Javed & Ahmed, 2022). Because of their health advantages, garlic scapes are used in food preparation and medicine. The presence of essential minerals such as calcium, phosphorus, iron, zinc, and selenium is noteworthy (Chen et al., 2019). Its basic nutritional profile makes it a rich source of antioxidants that aid in the body's fight against free radicals and many health benefits, including enhancing immune function, managing obesity, regulating lipid levels, fighting cancer inflammation, and combating respiratory disorders. These foods have properties that can assist in managing hypertension (Shang et al., 2019).

Garlic scape powder contains two potent antioxidants: flavonoids and polyphenols. These nutrients not only scavenge free radicals but also aid in lipid metabolism. These phytochemicals assist in the development of high‐density lipoprotein (HDL) and decrease low‐density lipoprotein (LDL) (Balogun & Kang, 2022). Obesity and other metabolic diseases such as cardiovascular diseases (CVD), hypertension, and diabetes can be caused by poor fat oxidation. Obesity has been found to have detrimental effects on lipid metabolism, thereby impacting the overall health of many organs, including the heart, kidney, and liver. Phytochemicals, including flavonoids and polyphenols, possess significant properties that can effectively enhance fat oxidation and sustain lipid metabolism in animal and human subjects (Liu et al., 2020). This study's primary aim is to evaluate the efficacy of garlic scape powder in lowering cholesterol levels in hyperlipidemic rats by incorporating garlic scape powder as a functional meal.

## 2 MATERIALS AND METHODS

### 2.1 Procurement of herbal material

Based on the alleged antioxidative properties of garlic scapes, components used for the current study were procured from the botanical market in Faisalabad, Pakistan, and then identified by a botanist under voucher no. 382‐bot‐23 and added in the herbarium in the Botany Department, Government College University, Faisalabad, Pakistan. All materials were dried and ground to powder.

### 2.2 Proximate analysis

Garlic scape powder was extensively analyzed to confirm the presence of biological components and secondary metabolites by following the previously described standard protocols (Ghalloo et al., 2022).

### 2.3 Phenolic acid profile by HPLC

The instrument consisted of the detector (SPD‐10AV), column C18 (25 cm × 4.6 mm, 5.0 μM), oven, and SIL‐20A autosampler (Shimadzu et al., Japan) was used to estimate the individual phenolic contents present in methanolic extract of *garlic scapes*. An amount of 0.1 g of extract was redissolved in 1 mL methanol, and a volume of 10 μL was injected through the HPLC system. The analytical method employed a linear gradient system, which consisted of (solvent A) 1% (v/v) acetic acid in water and (solvent B) ethanol. The gradient elution started with 15% for 0–15 min, 45% for 15–30 min, and 100% for 30–45 min. The flow rate was adjusted at 1 mL/min, and absorbance was detected at a wavelength of 280 nm. The analyte peaks were identified by matching their retention times and UV spectra with those of the reference standards (George et al., 2015).

### 2.4 Experimental trial and induction of hypocholesteremia by using a high‐fat diet

Adult albino rats (*n* = 60) weighing between 80 and 100 g were collected from the GCUF Department of Physiology's experimental station. Rats were kept in standardized environmental conditions of 25°C and 5% relative humidity. Rats were divided into six groups equally and randomly after 2 months of acclimatization. The nutrient composition of formulated food, such as protein, fiber, moisture, ash, and fat, was assessed using the standard AOAC (2006) method with minor modifications as described in Table 1.

**TABLE 1:** Composition of normal diet.

No	Dietary content	CMD (%)
1	Starch	76.00
2	Protein	10.00
3	Oil	10.00
4	Vitamin and mineral mixture	4.00

Abbreviation: CMD, chow maintains diet.

In an in vivo trial, normal healthy male Wistar albino rats (*n* = 60) aged 2 weeks were divided into a high‐fat diet group (HFD; *n* = 50) and a negative control group (NC; *n* = 10), as described in Table 2. All rats except the NC group were raised on an HFD (normal chow rodent diet mixed with 35% fat‐containing vegetable oil, cholic acid 0.5%, and cholesterol 1.25%) (Nisar et al., 2017) for 2 months until hyperlipidemia was confirmed (cholesterol level ≥ 250 mg/dL), whereas the NC group was fed with standard rodent chow diet (CMD). Hypercholesteremic rats were further divided randomly into five groups: positive control (PC; without any treatment), standard (STD; fenoget tablet containing fenofibrate 2 mg/kg bwt orally), G_1_ (garlic scape powder 400 mg/kg bwt), G_2_ (garlic scape powder 800 mg/kg bwt), and G_3_ (garlic scape powder 1200 mg/kg bwt) having same number of rats in each group (*N* = 10). All treatments were administered orally and continued for 3 months. The blood samples were examined for cholesterol, TG, HDL, LDL, apoprotein E, and albumin levels. The liver and kidney tissues of the rat were subjected to histopathology. All experiments were carried out with permission from the Government College University in Faisalabad's ethical review committee, using reference number GCUF/ERC/310.

**TABLE 2:** Experimental groups.

Group	Treatment
NC	CMD (chow rodent diet) throughout the experimental period
PC	HFD throughout the experimental period HFD; normal chow rodent diet mixed with 35% fat‐containing vegetable oil, cholic acid 0.5%, and cholesterol 1.5%
STD	HFD + fenoget (20 mg/kg but/day orally) throughout the experimental period
G_1_	HFD + powder form of garlic scapes (400 mg/kg)
G_2_	HFD + powder form of garlic scapes (800 mg/kg)
G_3_	HFD + powder form of garlic scapes (1200 mg/kg)

Abbreviations: G1, treatment 1 (garlic scape powder 400 mg/kg bwt); G2, treatment 2 (garlic scape powder 800 mg/kg bwt); G3, treatment 3 (garlic scape powder 1200 mg/kg bwt); HFD, high‐fat diet; NC, normal control; PC, positive control; STD, standard control group.

### 2.5 Serum lipid profile

Lipid profiles are blood tests that measure lipid levels in the blood, such as cholesterol and TG, and provide details on cardiovascular risk and some genetic illnesses. Total cholesterol, HDL, LDL, and total TG are among its components. The animals in the experimental paradigm were fasted for 12 h on the last day before anesthetics were administered and blood samples were taken. When blood is drawn after a meal, dietary fat may enter the bloodstream, giving rise to the idea of a misleading cholesterol level. It is essential to take blood samples following the fasting period to ensure a true reflection of the serum cholesterol status and an accurate assessment of cholesterol levels.

### 2.6 Total cholesterol (TC)

The TC levels in blood serum were estimated using colorimetric test kits (Sigma‐Aldrich, Germany, catalog number C$0005; mg/dL; range of measurement: 1–5 μg). Briefly, 50 μL of the prepared cholesterol reagent was added to sample filled 96‐well microtiter plates, and the contents were mixed thoroughly. Then, the prepared sample tubes were placed in a dark room at 37°C for 30 min. The absorbance of the sample was measured at a wavelength of 546 nm against the blank reagent. On oxidation and enzymatic hydrolysis, the total cholesterol content in serum is measured. Quinonimine, which is produced via Trinder's reaction between 4‐amino antipyrine and phenol, acted as a colorimetric indicator due to the catalytic activity of peroxidase (Mustafa et al., 2022).

### 2.7 Triglycerides

The content of TG in serum was measured using commercially available colorimetric assay kits made by Sigma‐Aldrich, Germany (catalog number MAK266; mg/dL; reactivity: 2 pmol–10 mol; range of measurement: 2–10,000 JM range). Based on lipoprotein lipase's enzymatic breakdown of blood TG, the concentration of serum TG is measured. In a reaction catalyzed by peroxidase, hydrogen peroxide combines with 4‐amino antipyrine and 4‐chlorophenol to produce quinonimine, which serves as a colorimetric indicator (Mustafa et al., 2022).

### 2.8 High‐density lipoprotein‐cholesterol (HDL‐Chol)

HDL‐Chol was calculated using the HDL phosphotungstic precipitation method by a bioresearch kit (catalog number CS009 1100). After thoroughly combining all ingredients, the mixture was allowed to sit at room temperature for 10–15 min. In the end, the absorbance of all samples was measured against the blank reagent for 60 min at a wavelength of 546 nm. The mixture was gently stirred and allowed to sit at room temperature for 10–15 min.

### 2.9 Low‐density lipoprotein‐cholesterol (LDL‐Chol)

The serum levels of LDL were calculated using the following Friedrick equation:

(1) $$\mathrm{LDL}-\text{cholesterol}=\left(\text{total cholesterol}\right)–\left(\text{triglyceride}/5\right)–\left(\mathrm{HDL}-\text{cholesterol}\right)$$

### 2.10 Apoprotein E

According to the manufacturer's guidelines, the ELISA kit method measured the apoprotein E level. ELISA kit Rat ApoE (apoprotein E) was purchased from Elabscience, catalog number E‐EL‐R1230 with lot number 9AHCC5VPH having a detection range of 3.13–200 ng/mL, sensitivity 1.88 ng/mL, cv <10%.

### 2.11 Serum insulin

Serum insulin was determined at the end of treatment on the 28th day. A commercially available ELISA kit (catalog number E‐EL‐R2466) was purchased from E‐Lab Sciences, showing the sensitivity of 0.47 μU/mL with a detection range of 0.78–50 ng/mL and CV% <10 was used to estimate serum insulin.

### 2.12 Serum liver enzymes

#### 2.12.1 Alanine aminotransferase (ALT)

The kit method was used to measure the serum ALT level (ALT (GPT) SR) by Bioactive (catalog number 10498 99 93183). Briefly, a 100 μL of sample, was added to 500 μL of R 1 buffer and kept at 37°C for 30 min. A tube of distilled water was used as a reagent blank, which was tested alongside the standard and samples. Next, 500 μL of reagent R 2 was pipetted into each tube and kept at 25°C for 20 min. After the second incubation, the reaction was stopped by adding diluted R 3 (1:10 R 3 by distilled water). The absorbance of samples and standards was measured (Liu et al., 2014).

#### 2.12.2 Aspartate aminotransferase test (AST)

AST in the serum was measured using a commercially available liquiform method kit supplied by AST (Crescent® Diagnostic Kit, Jeddah, catalog number 15204C). A 100 μL sample was gently combined with 500 μL buffer R1 and vortexed. After 30 min of incubation at 37°C, a reagent blank (100 μL distilled water) was utilized. Then, 500 μL of reagent R2 was pipetted into an Eppendorf tube and incubated for 20 min at 25°C. The reaction was stopped by adding 5.0 mL of R3 (sodium hydroxide), which was then incubated for an additional 5 min. The sample absorbance was then measured at a wavelength of 546 nm against the reagent blank (Schemitt et al., 2016).

### 2.13 Histopathology

The liver and pancreatic tissues were removed and fixed for 24 h in 10% paraformaldehyde before being subjected to varying ethanol concentrations to dehydrate them. Following tissue fixation, tissue dehydration was carried out by passing it through different concentrations of alcohol, while xylene was used as a clearing agent at different periods. Following infiltration and embedding, sectioning was carried out using a microtome. Slides were properly cleaned during mounting. Afterward, the tissues were fixed in paraffin wax and cut using a microtome into thin slices of 2–4 μm in diameter. Hematoxylin and eosin stains were subsequently applied to the deparaffinized sections. A light microscope examined the stained slices (Sheng et al., 2020).

### 2.14 Statistical analysis

Data sets collected from different parameters were presented in mean ± SEM. A two‐way analysis of variance was applied using the Prism 9.2.0 software for the evaluation of significant difference (p≤0.05). The Duncan Multiple Range (DMR) test was applied in Costas 2.0 to determine the significant variation (Montgomery, 2017).

## 3 RESULTS

### 3.1 Chemical composition and concentration of bioactive compounds in garlic scapes

The proximate analysis of the crude dry powder of garlic scapes is shown in Table 3. Carbohydrates (35.26%), proteins (21.00%), and crude fiber (8.23%) were found in higher percentages; however, crude fat (14.13%) and ash content (11.99%) were also determined, while energy harvest was 299.11 K calories/100 g of dry powder as shown in Table 3.

**TABLE 3:** Percentages of different parameters and materials found in garlic scapes/100 g dry powder.

Parameters	Result
Moisture	9.39 ± 0.3^e^
Ash	11.99 ± 0.6^d^
Crude fat	8.23 ± 0.4^e^
Protein (N*6.25)	21 ± 0.9^b^
Carbohydrates	35.26 ± 1.1^a^
Crude fiber	14.13 ± 0.6^c^
K calories/100 g	299.11

Superscript letters a‐e represents different significance.

**FIGURE 1:**
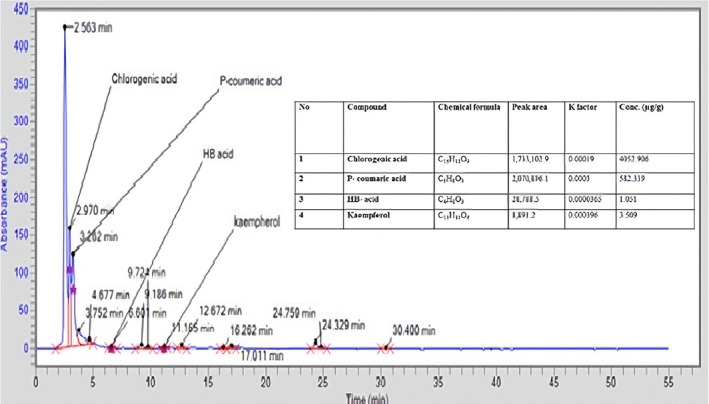
Quantitative analysis of various bioactive phenolic and flavonoid compounds in garlic scape powder by HPLC‐UV chromatogram.

High‐performance liquid chromatographic analysis of garlic scape powder revealed the presence of four phenolic compounds: chlorogenic acid (4052.906 μg/g), *p*‐coumaric acid (582.339 μg/g), hydroxybenzoic acid (HB) acid (1.051 μg/g), and kaempferol (3.509 μg/g). Chlorogenic acid was found with maximum concentration, whereas HB acid was found with minimum concentration, as depicted in Figure 1.

### 3.2 Body weight

**FIGURE 2:**
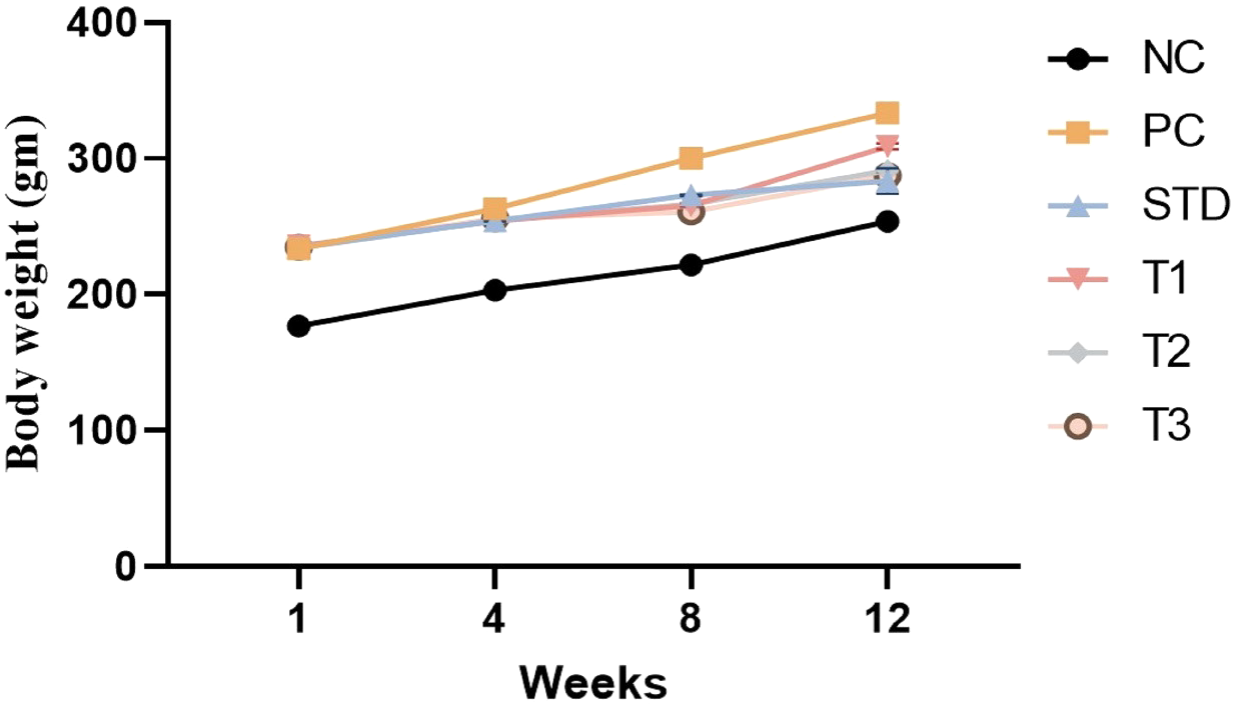
Body weight in the NC (normal control), PC (positive control), STD group (fenoget oral dose of 20 mg/kg bwt), and different garlic scape powder‐treated groups.

Animal body weight (g) in the NC, PC, STD control, and treatment groups was measured. The overall body weight (mean ± SEM) decreased significantly (*p* ≤ .05) in the treatment groups compared to other groups except for NC (Table 4). The body weight was significantly reduced (*p* ≤ .05) in the STD (261.42 ± 1.13 g), G_1_ (266.12 ± 1.58 g), G2 (261.57 ± 0.73 g), and G3 groups (259.7 ± 0.31 g) compared to the PC group (282.62 ± 1.44 g) as shown in Figure 2.

**TABLE 4:** Body weight (g ± SEM) in the NC, PC, STD group (fenoget oral dose of 20 mg/kg bwt), and different doses of garlic scape powder‐treated groups.

Group	First week	Fourth week	Eighth week	Twelfth week	Overall mean
NC	176.8 ± 1.11	203.1 ± 1.19	221.9 ± 1.03	253.8 ± 1.75	213.9 ± 1.27
PC	233.4 ± 1.13	263.3 ± 1.61	300.2 ± 2.01	333.6 ± 1.01	282.62 ± 1.44
STD	234.9 ± 0.83	254.2 ± 0.62	273 ± 1.09	283.6 ± 1.98	261.42 ± 1.13
G_1_	235.9 ± 2.75	254.2 ± 1.05	265.5 ± 1.27	308.9 ± 1.25	266.12 ± 1.58
G_2_	234.3 ± 0.82	254.4 ± 0.25	266.5 ± 0.47	291.1 ± 1.53	261.57 ± 0.73
G_3_	234.8 ± 0.39	255.7 ± 0.28	260.7 ± 0.14	287.6 ± 0.45	259.7 ± 0.31

Abbreviations: G1, treatment 1; G2 = treatment 2; G3, treatment 3; NC, normal control; PC, positive control; STD, standard control group (fenoget oral dose of 20 mg/kg bwt).

### 3.3 Total cholesterol (TC)

The overall mean TC (mean ± SEM) significantly (*p* ≤ .05) decreased in the treatment groups compared to all other groups except NC. TC showed a significant (*p* ≤ .05) decrease in the STD group (100.45 ± 0.71 mg/dL) and in the treatment groups G_1_ (96.95 ± 0.54 mg/dL), G_2_ (95.67 ± 1.28 mg/dL), and G_3_ (88.1 ± 0.85 mg/dL) compared to the PC group (114.7 ± 0.85 mg/dL).

**FIGURE 3:**
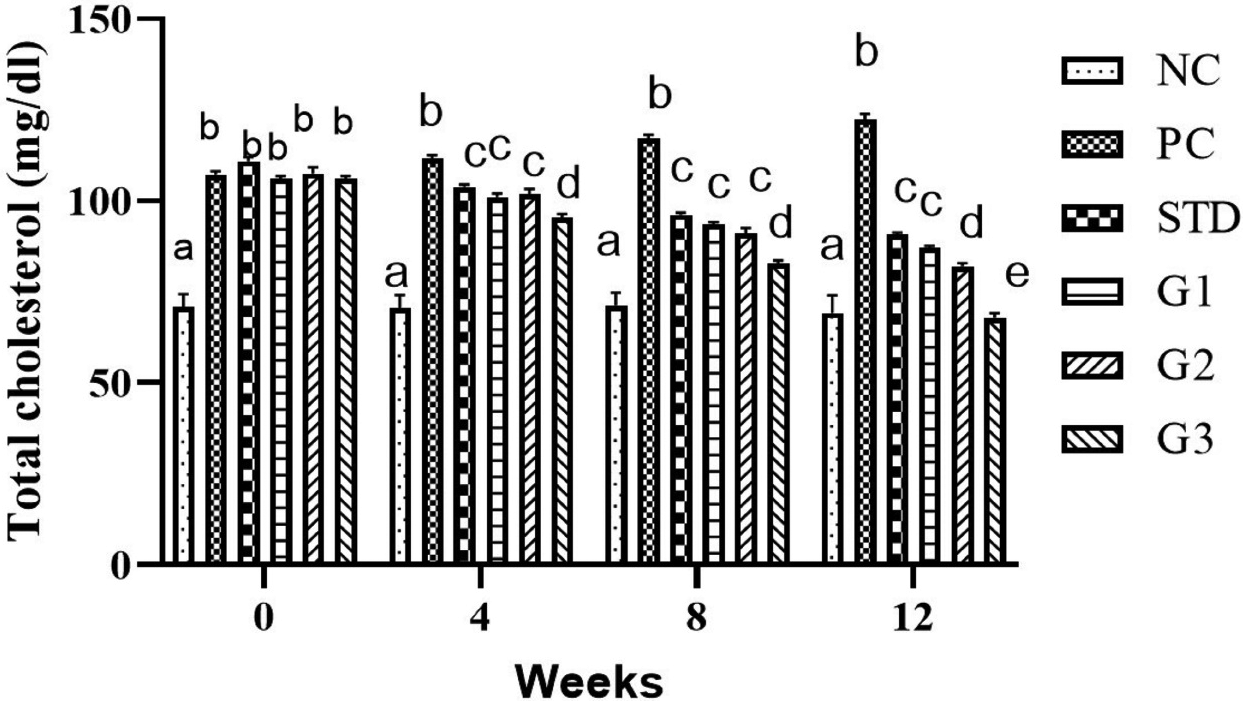
Cholesterol levels in NC (normal control), PC (positive control), STD group (fenoget oral dose of 20 mg/kg bwt), and different garlic scape powder‐treated groups: Group 1 (400 mg/kg bwt), Group 2 (800 mg/kg bwt), and Group 3 (1200 mg/kg bwt) for 3 months in HFD‐induced dyslipidemic rats. *N = 10. a‐e Means with different superscripts show significant difference (p ≤ .05).

At zero week, there was a nonsignificant (*p* ≤ .05) decrease in cholesterol levels in all groups except NC. In the fourth and eighth weeks, the cholesterol level was significantly (*p* ≤ .05) higher in the PC group than in all other groups. At the 12th week, the cholesterol level significantly (*p* ≤ .05) decreased in the STD group (90.8 ± 0.44 mg/dL) and in the treatment groups G_1_ (87.2 ± 0.46 mg/dL), G_2_ (82.1 ± 0.83 mg/dL), and G_3_ (67.9 ± 1.17 mg/dL) compared to the PC group (122.4 ± 1.48 mg/dL) as shown in Figure 3.

### 3.4 Triglyceride

The overall mean total TG significantly (*p* ≤ .05) decreased in the treatment groups compared to all other groups except NC. Total TG showed a significant (*p* ≤ .05) decrease in the STD group (112.7 ± 2.39 mg/dL) and in the treatment groups G_1_ (113.72 ± 1.35 mg/dL), G_2_ (109.52 ± 1.43 mg/dL), and G_3_ (107.87 ± 1.08 mg/dL) compared to the PC group (127.85 ± 1.95 mg/dL).

**FIGURE 4:**
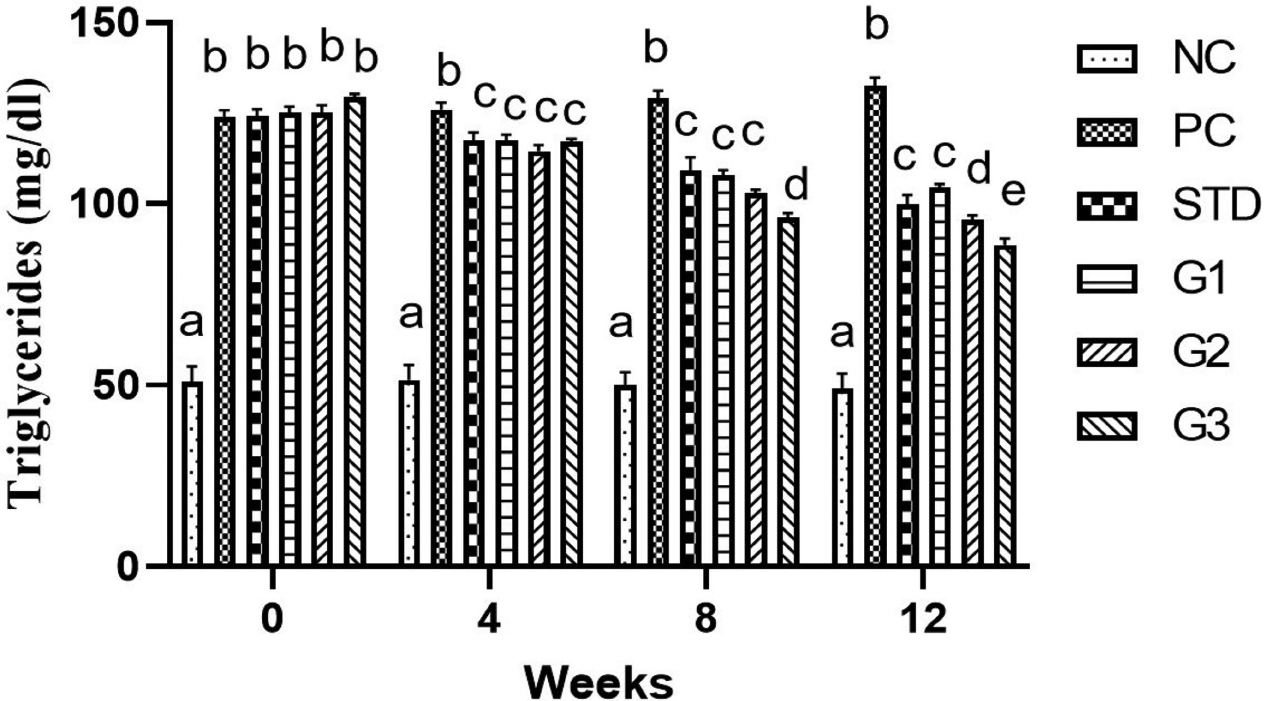
Levels of triglycerides in rats with high‐fat diet (HFD) and three groups treated with garlic scape powder: Group 1 (400 mg/kg bwt), Group 2 (800 mg/kg bwt), and Group 3 (1200 mg/kg bwt) for 3 months. The *p*‐value for this comparison was 0.05, and there were 10 rats in each group.

At zero week, there was a nonsignificant (*p* ≤ .05) decrease in TG levels in all groups except NC. In the fourth and eighth weeks, the TG level was significantly (*p* ≤ .05) higher in PC than in all other groups. At the 12th week, the TG level significantly (*p* ≤ .05) decreased in the STD group (99.9 ± 2.37 mg/dL) and in the treatment groups G_1_ (104.4 ± 0.94 mg/dL), G_2_ (95.5 ± 1.22 mg/dL), and G_3_ (88.7 ± 1.64 mg/dL) compared to PC group (132.6 ± 2.08 mg/dL) as shown in Figure 4.

### 3.5 High‐density lipoprotein (HDL)

**FIGURE 5:**
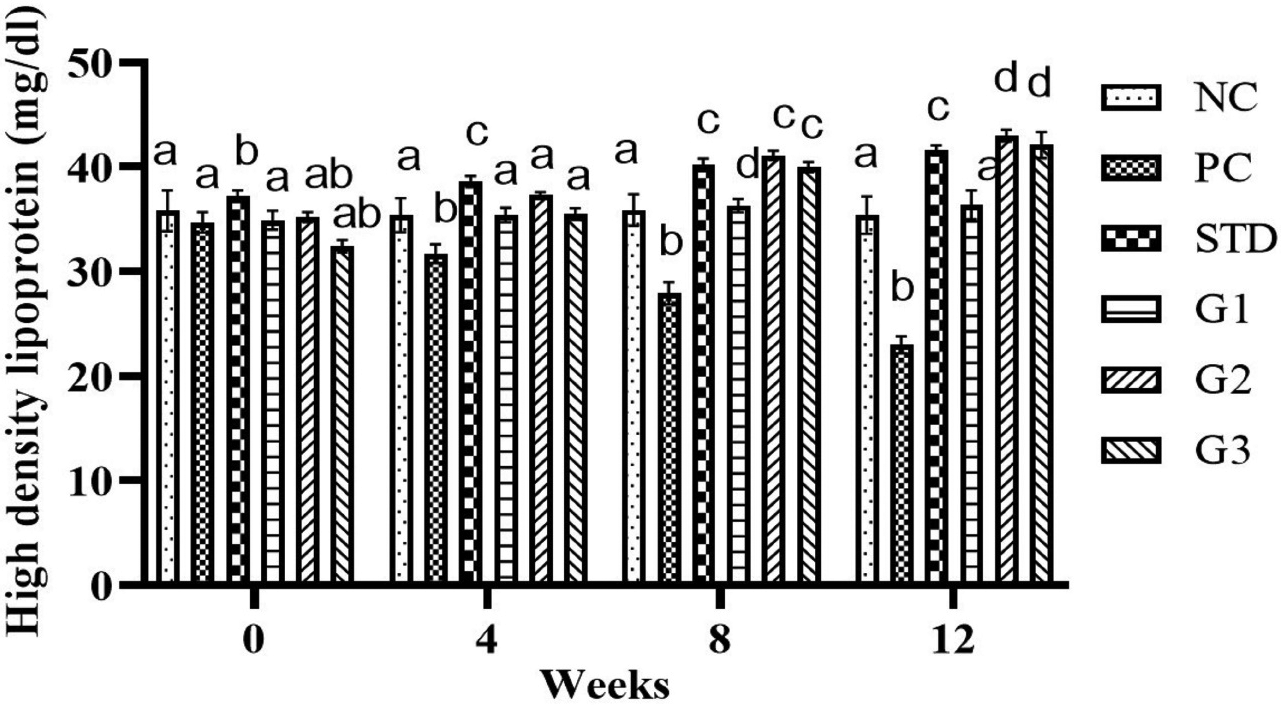
High‐density lipoprotein levels in the NC (normal control), PC (positive control), STD group (fenoget oral dose of 20 mg/kg bwt), and different garlic scape powder‐treated groups: Group 1 (400 mg/kg bwt), Group 2 (800 mg/kg bwt), and Group 3 (1200 mg/kg bwt) for 3 months in HFD‐induced dyslipidemic rats, considering *p* ≤ .05. *N = 10.

The overall mean HDL significantly (*p* ≤ .05) increased in the treatment groups compared to all other groups except NC. HDL showed a significant (*p* ≤ .05) decrease in the STD group (112.7 ± 2.39 mg/dL) and in the treatment groups G_1_ (33.75 ± 0.86 mg/dL), G_2_ (39.15 ± 0.42 mg/dL), and G_3_ groups (37.5 ± 0.87 mg/dL) compared to the PC group (29.32 ± 0.88 mg/dL). At zero week, there was a nonsignificant (*p* ≤ .05) change in HDL levels in all groups except NC. In the fourth and eighth weeks, HLD significantly (*p* ≤ .05) reduced in PC compared to all other groups. At the 12th week, the HDL level significantly (*p* ≤ .05) increased in STD group (41.6 ± 0.45 mg/dL) and in the treatment groups G_1_ (36.4 ± 1.31 mg/dL), G_2_ (43 ± 0.54 mg/dL), and G_3_ (42.1 ± 1.81 mg/dL) compared to PC group (23 ± 0.76 mg/dL) as shown in Figure 5.

### 3.6 Low‐density lipoprotein (LDL)

**FIGURE 6:**
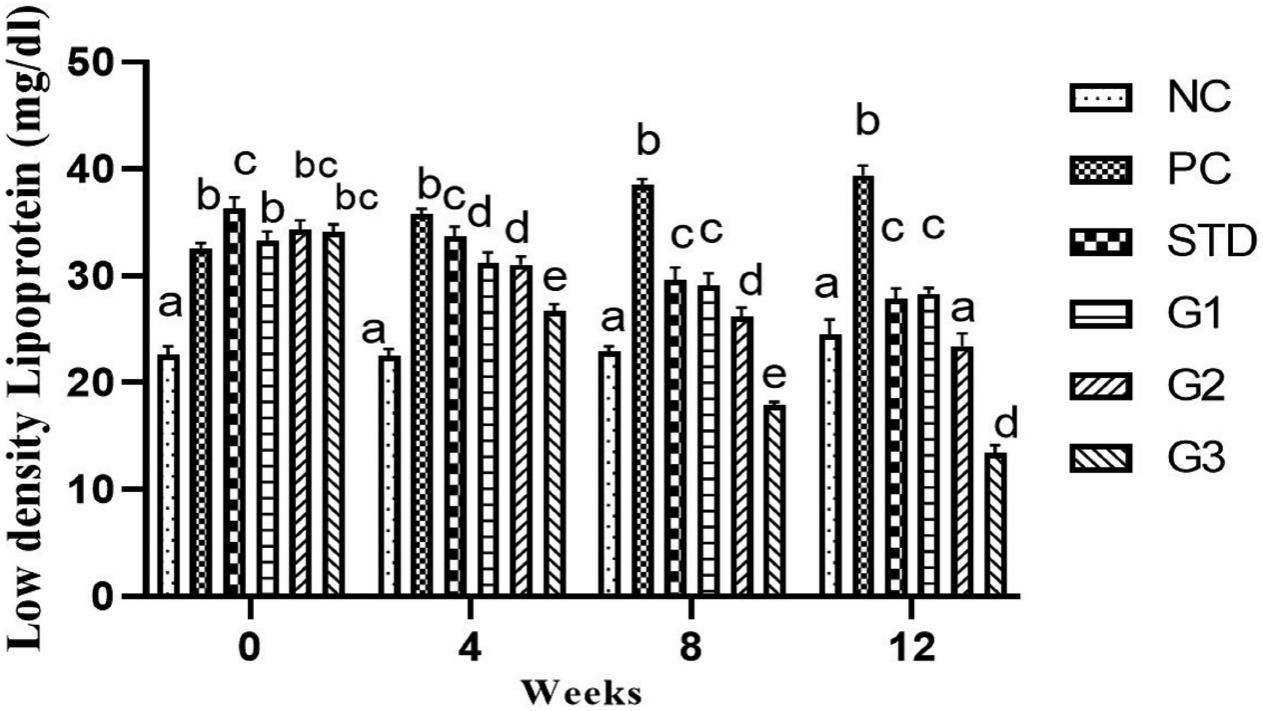
Low‐density lipoprotein levels in NC (normal control), PC (positive control), STD group (fenoget oral dose of 20 mg/kg bwt) and different garlic scape powder‐treated groups: Group 1 (400 mg/kg bwt), Group 2 (800 mg/kg bwt), and Group 3 (1200 mg/kg bwt) for a 3‐month period in HFD‐induced dyslipidemic rats, considering *p* ≤ .05. *N = 10.

The overall mean LDL significantly (*p* ≤ .05) decreased in the treatment groups compared to all other groups except NC. LDL protein showed a significant (*p* ≤ .05) decrease in the STD group (31.85 ± 0.98 mg/dL) and in the treatment groups G_1_ (30.47 ± 0.83 mg/dL), G_2_ (28.72 ± 0.89 mg/dL), and G_3_ (37.92 ± 0.86 mg/dL) compared to the PC group (36.55 ± 0.59 mg/dL). At zero week, there was a nonsignificant (*p* ≤ .05) decrease in LDL levels in all groups except NC. In the fourth and eighth weeks, LDL levels were significantly (*p* ≤ .05) higher in PC than in all other groups. In the 12th week, LDL level significantly (*p* ≤ .05) decreased in the STD group (27.8 ± 0.95 mg/dL) and in the treatment groups G1 (28.3 ± 0.53 mg/dL), G2 (23.4 ± 1.15 mg/dL), and G3 (12.1 ± 1.81 mg/dL) compared to the PC group (39.4 ± 0.87 mg/dL) as shown in Figure 6.

### 3.7 Apoprotein E

**FIGURE 7:**
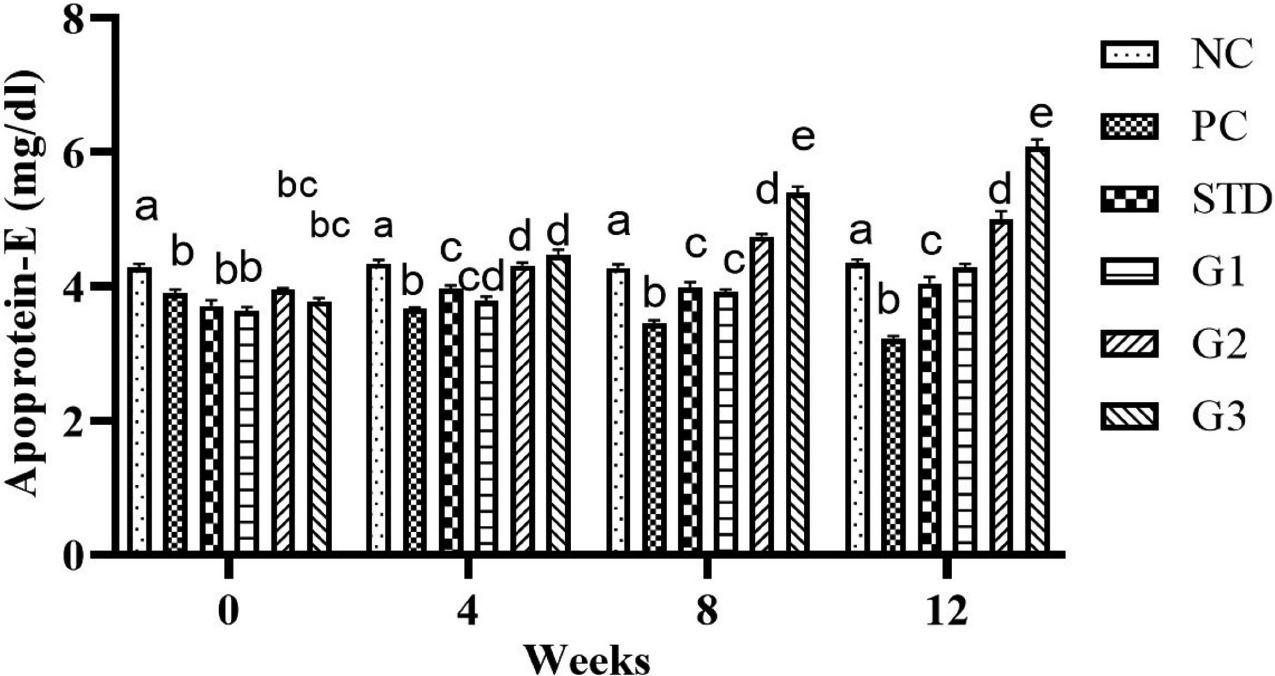
Apoprotein E levels in NC (normal control), PC (positive control), STD group (fenoget oral dose of 20 mg/kg bwt) and different garlic scape powder‐treated groups: Group 1 (400 mg/kg bwt), Group 2 (800 mg/kg bwt), and Group 3 (1200 mg/kg bwt) for 3 months in HFD‐induced dyslipidemic rats considering *p* ≤ .05. *N = 10.

The overall mean apoprotein E significantly (*p* ≤ .05) increased in the treatment groups compared to all other groups except NC. Apoprotein E showed a significant (*p* ≤ .05) increase in the STD group (3.92 ± 0.07) and in the treatment groups G_1_ (3.90 ± 0.04), G_2_ (4.5 ± 0.04), and G_3_ (4.89 ± 0.07) compared to the PC group (3.55 ± 0.03). At zero week, there was a nonsignificant (*p* ≤ .05) increase in apoprotein E levels in all groups except NC. In the fourth and eighth weeks, apoprotein E level significantly (*p* ≤ .05) reduced in PC compared to all other groups. At the 12th week, apoprotein E level significantly (*p* ≤ .05) increased in the STD group (4.04 ± 0.09) and in the treatment groups G1 (4.28 ± 0.04), G2 (5.00 ± 0.11), and G3 (6.08 ± 0.10) compared to PC (3.22 ± 0.03) as shown in Figure 7.

### 3.8 Serum insulin

**FIGURE 8:**
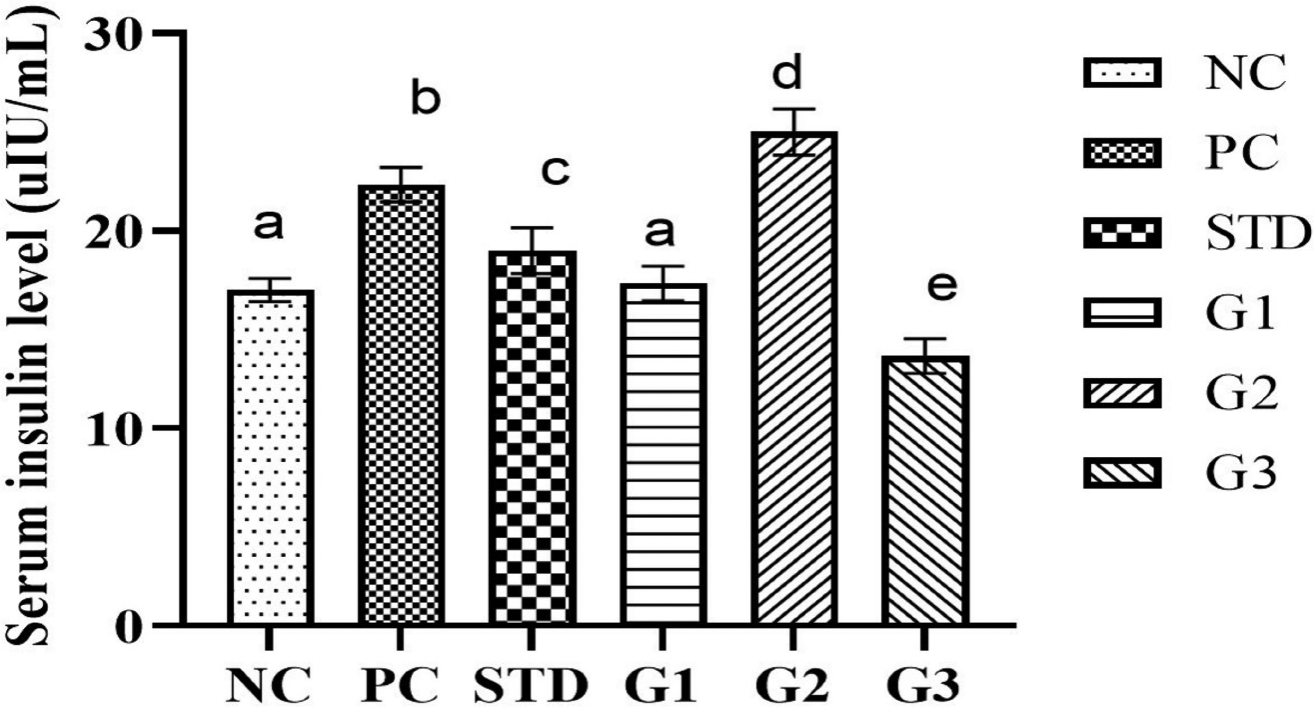
Serum insulin levels at different weeks (0, 4, 8, and 12) in NC (normal control), PC (positive control), STD group (fenoget oral dose of 20 mg/kg bwt), and different garlic scape powder‐treated groups: Group 1 (400 mg/kg bwt), Group 2 (800 mg/kg bwt), and Group 3 (1200 mg/kg bwt) orally administered for 3 months in HFD‐induced dyslipidemic rats considering *p* ≤ .05. Dissimilar superscripts are statistically significant with each other. *N = 10.

The overall serum insulin significantly decreased (*p* ≤ .05) in the treatment groups (G1 and G3) compared to all other groups except NC. The serum insulin level showed a significant decrease in the STD group (19 ± 0.95 μL/mL) and in the treatment groups G_1_ (17.33 ± 0.72 μL/mL) and G_3_ (13.66 ± 0.72 μL/mL) compared to G_2_ (25 ± 0.94 μL/mL) and PC (22.33 ± 0.72 μL/mL). In PC and G_2_, serum insulin significantly (*p* ≤ .05) increased compared to all other groups, as shown in Figure 8.

### 3.9 Alanine transaminase (ALT)

The overall mean ALT significantly (*p* ≤ .05) decreased in the treatment groups compared to all other groups except NC. ALT (U/L) showed a significant (*p* ≤ .05) decrease in STD group (77.5 ± 1.75 U/L) and in the treatment groups G1 (76.32 ± 1.49 U/L), G2 (72.02 ± 0.87 U/L), and G3 (67.3 ± 1.41 U/L) compared to PC (88.87 ± 1.47 U/L).

**FIGURE 9:**
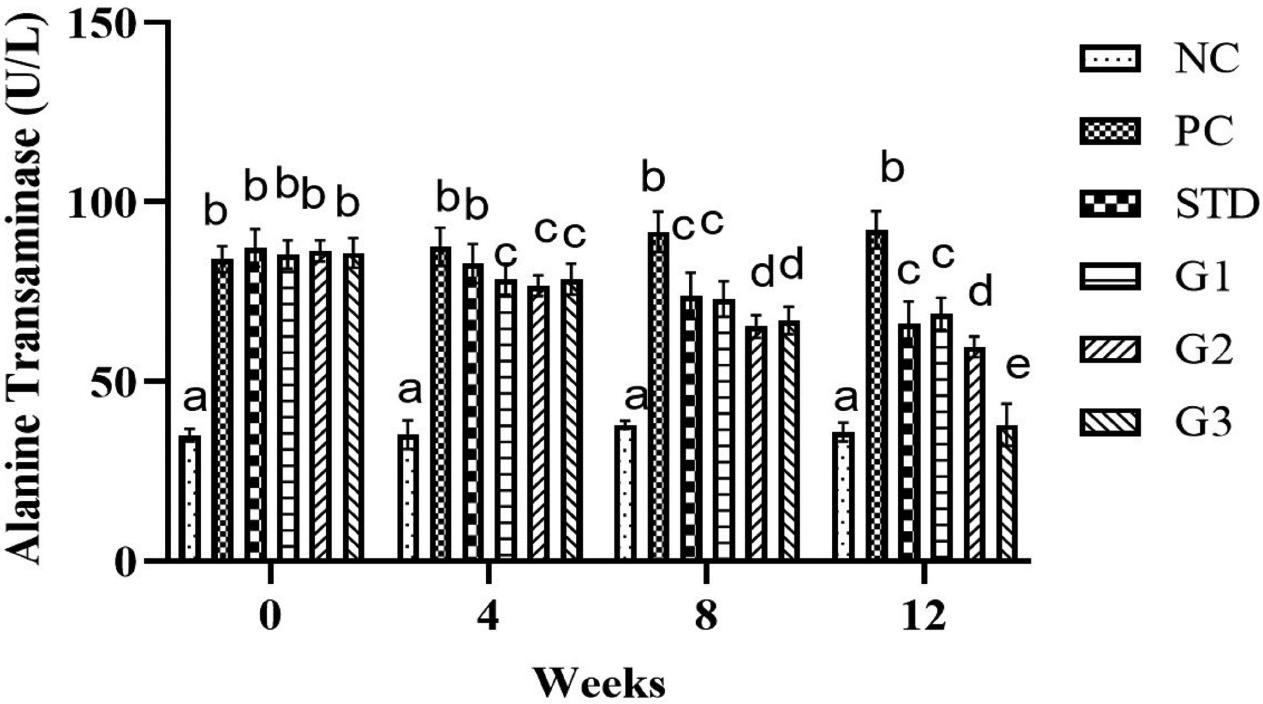
Alanine transaminase levels in NC (normal control), PC (positive control), STD group (fenoget oral dose of 20 mg/kg bwt), and different garlic scape powder‐treated groups: Group 1 (400 mg/kg bwt), Group 2 (800 mg/kg bwt), and Group 3 (1200 mg/kg bwt) for 3 months in HFD‐induced dyslipidemic rats, considering *p* ≤ .05. *N = 10.

At zero week, there was a nonsignificant (*p* ≤ .05) decrease in ALT (U/L) levels in all groups except NC. In the fourth and eighth weeks, the ALT (U/L) level was significantly (*p* ≤ .05) high in PC compared to all other groups. At the 12th week ALT (U/L) level significantly (*p* ≤ .05) decreased in the STD group (66 ± 1.88 U/L) and in the treatment groups G_1_ (68.9 ± 1.32 U/L), G_2_ (59.7 ± 0.83 U/L), and G_3_ (37.9 ± 1.76 U/L) compared to PC (92.2 ± 1.55 U/L) as shown in Figure 9.

### 3.10 Aspartate aminotransferase (AST)

**FIGURE 10:**
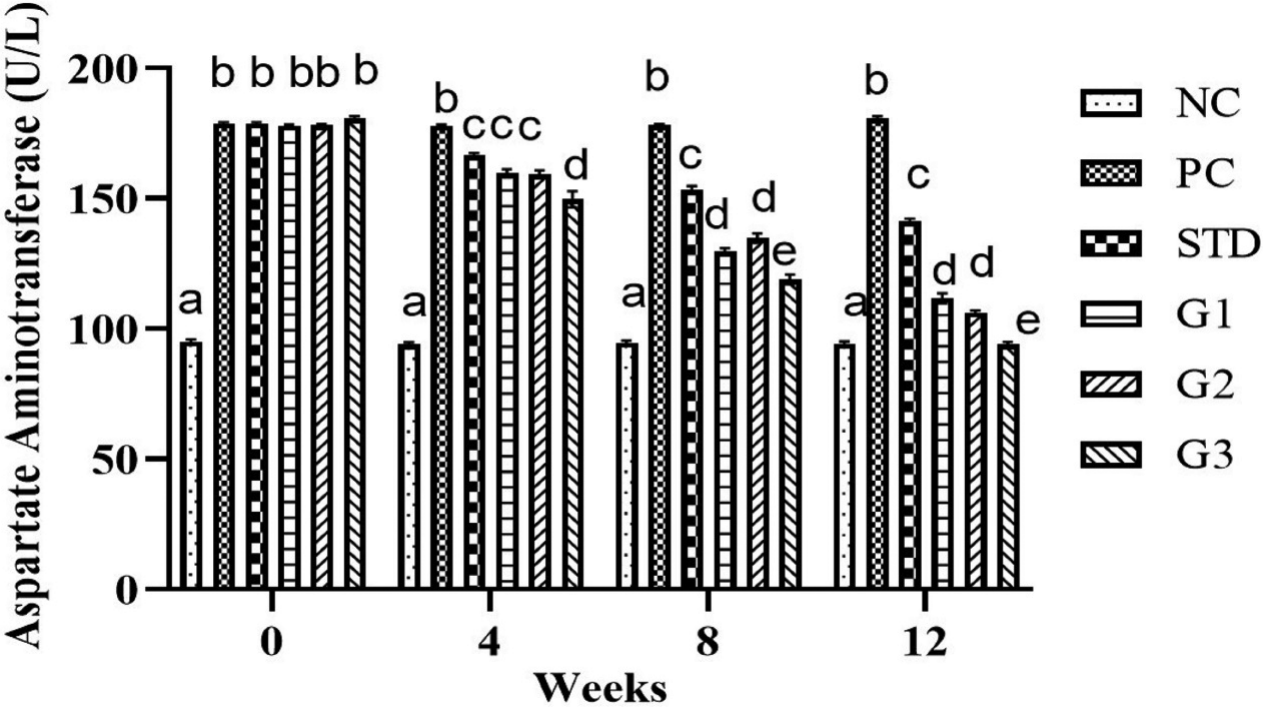
Aspartate aminotransferase levels in the NC (normal control), PC (positive control), STD group (fenoget oral dose of 20 mg/kg bwt) and different garlic scape powder‐treated groups: Group 1 (400 mg/kg bwt), Group 2 (800 mg/kg bwt), and Group 3 (1200 mg/kg bwt) for 3 months in HFD‐induced dyslipidemic rats, considering *p* ≤ .05. *N = 10.

The overall mean AST significantly (*p* ≤ .05) decreased in the treatment groups compared to all other groups except NC. AST showed a significant (*p* ≤ .05) decrease in the STD group (159.82 ± 1.01 U/L) and in the treatment groups G1 (144 ± 1.22 U/L), G2 (144.6 ± 1.31 U/L), and G3 (135.87 ± 1.56 U/L) compared to PC (178.9 ± 0.54 U/L). At zero week, there was a nonsignificant (*p* ≤ .05) decrease in AST levels in all groups except NC. In the fourth and eighth weeks, the AST level was significantly (*p* ≤ .05) high in PC compared to all other groups. At the 12th week, AST level significantly (*p* ≤ .05) decreased in the STD group (141 ± 1.11 U/L) and in the treatment groups G1 (111.7 ± 1.80 U/L), G2 (106.2 ± 0.70 U/L), and G3 (94.1 ± 0.74 U/L) compared to PC (180.8 ± 0.79 U/L) as shown in Figure 10.

### 3.11 Albumin

**FIGURE 11:**
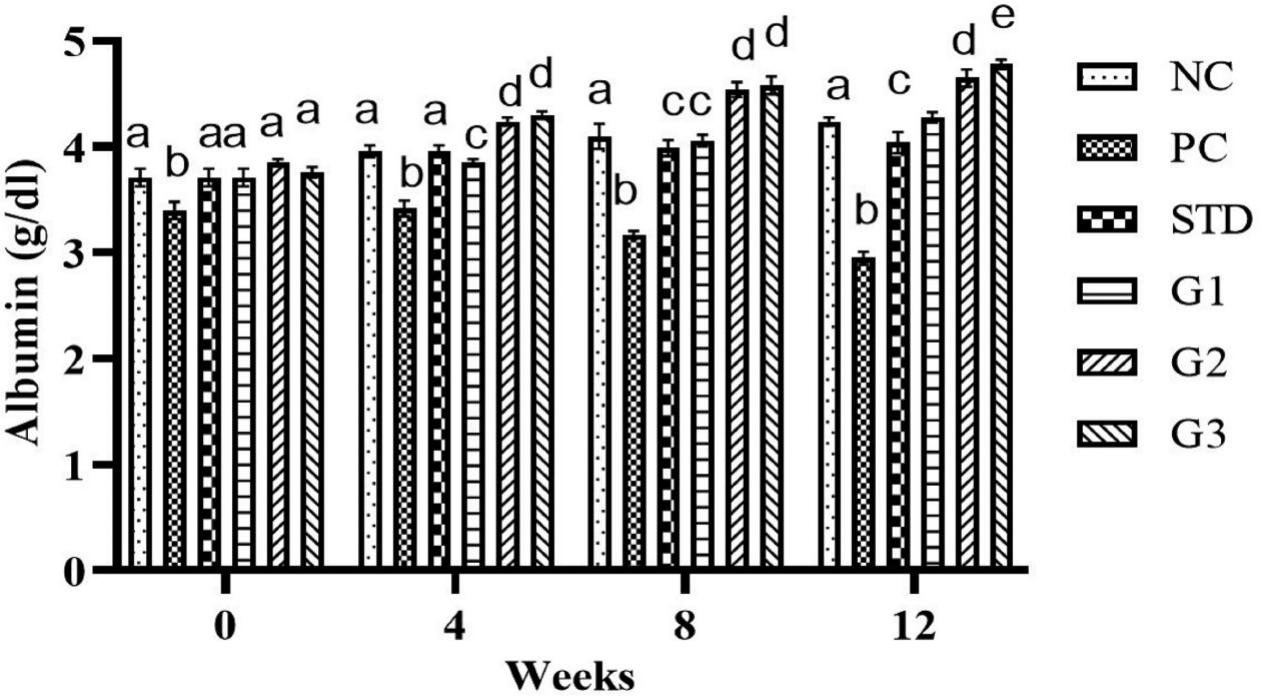
Albumin (g/dL ± SEM) levels in NC (normal control), PC (positive control), STD group (fenoget oral dose of 20 mg/kg bwt) and different garlic scape powder‐treated groups: Group 1 (400 mg/kg bwt), Group 2 (800 mg/kg bwt), and Group 3 (1200 mg/kg bwt) for 3 months in HFD‐induced dyslipidemic rats considering *p* ≤ .05. *N = 10.

The overall mean albumin significantly (*p* ≤ .05) increased in the treatment groups compared to all other groups except NC. Albumin showed a significant (*p* ≤ .05) increase in the STD group (12.67 ± 0.06) and in the treatment groups G1 (12.69 ± 0.16), G2 (13.79 ± 0.13), and G3 (13.83 ± 0.15) compared to the PC group (10.72 ± 0.18). At zero week, there was a nonsignificant (*p* ≤ .05) increase in albumin levels in all groups except NC. In the fourth and eighth weeks, the albumin level significantly (*p* ≤ .05) reduced in PC compared to all other groups. In the 12th week, the albumin level significantly (*p* ≤ .05) increased in the STD group (4.04 ± 0.09) and in the treatment groups G1 (4.28 ± 0.04), G2 (4.65 ± 0.07), and G3 groups (4.78 ± 0.04) compared to the PC group (2.95 ± 0.05) as shown in Figure 11.

### 3.12 Histopathology

**FIGURE 12:**
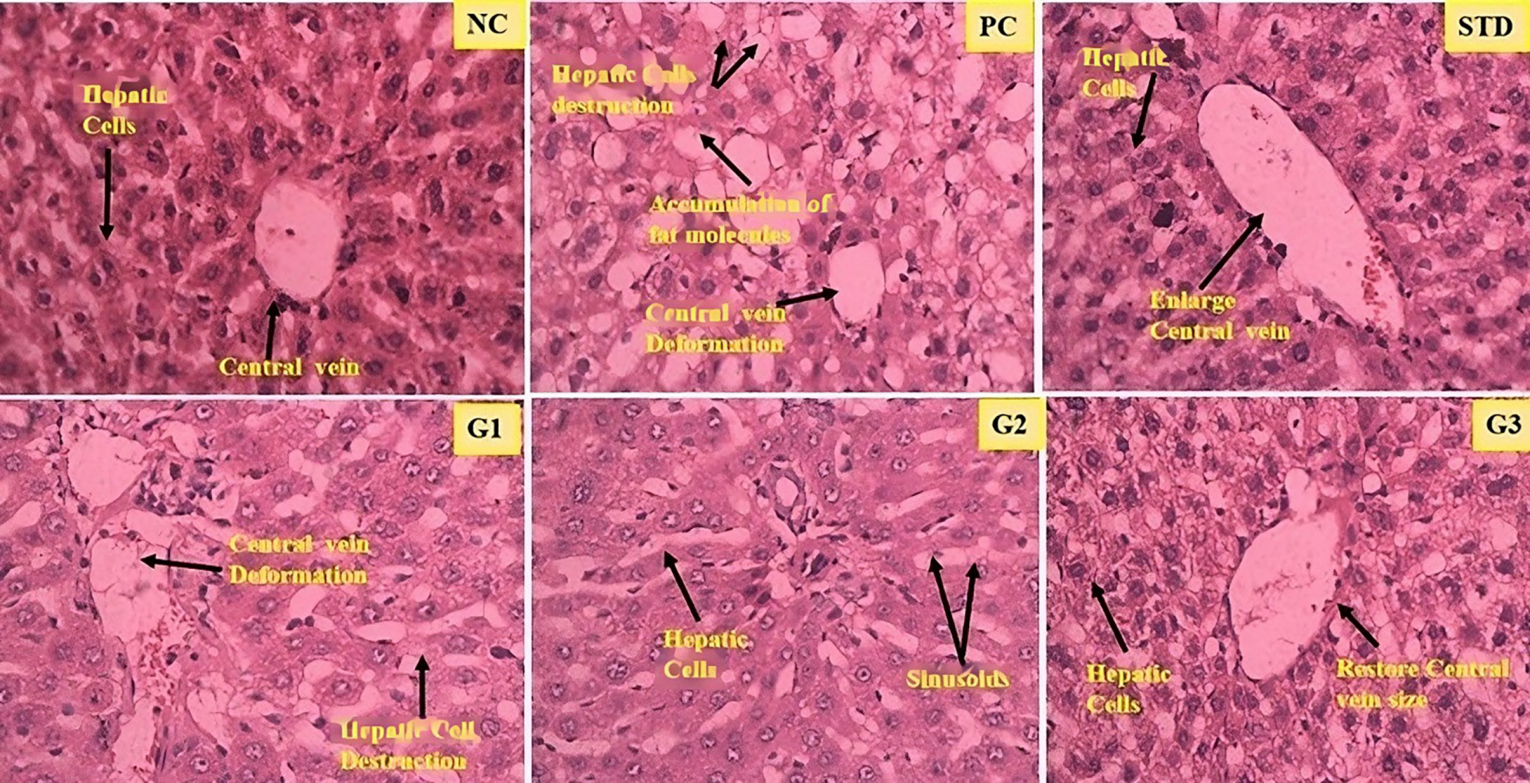
A photomicrograph shows the changes in the histopathology of the NC (normal control), PC (positive control), STD group (fenoget oral dose of 20 mg/kg bwt), and three groups of rats that were treated with garlic scape powder for 3 months: Group 1 (400 mg/kg bwt), Group 2 (800 mg/kg bwt), and Group 3 (1200 mg/kg bwt). The groups were all given different amounts of fenoget.

The liver tissues from the NC were looked at and found to have normal‐sized hepatocytes portal veins and nuclei (A). However, in the PC and G1 (400 mg/kg bwt) groups, the hepatocytes were dead, the cell structures were scattered, and the shape of the portal vein was altered. This meant that the cells lost their contents and their integrity. Rats in the STD (fenofibrate, 20 mg/kg bwt) group had liver tissue with a portal vein approximately the same size as usual, but the structure was abnormal. The treated groups, G2 (800 mg/kg bwt) and G3 (1200 mg/kg bwt), regained normal histological structures, normal hepatic parenchyma, portal vein size, and well‐organized nuclei, in contrast to the PC group (Figure 12).

## 4 DISCUSSION

In the current study, rats of different groups exhibited a remarkably higher body weight increase due to an HFD administered to induce hypocholesteremia. An HFD of 35% vegetable oil (Mustafa et al., 2022) along with a normal CMD. Qushawy et al. (2022) increased high‐energy harvest and storage (Uba et al., 2023). The garlic scape‐treated groups maintained their body weight at Weeks 8 and 12 (Moon & Koh, 2020). High protein content at different dose levels of garlic scape (*Allium sativum* family) powder leads to a sustained reduction in body weight due to reduced appetite and less intake, as reported earlier (Tokushige et al., 2021). In the PC hypercholesterolemic group, gut‐derived hormones ghrelin made them hungrier, whereas glucagon‐like peptide‐1 (GLP‐1) and cholecystokinin made them less hungry (Abdel‐Daim et al., 2020). By boosting the expression of uncoupling protein mRNA and activating AMP‐activated protein kinase, dietary garlic consumption lowers body weight and is associated with diet‐induced obesity (Taha et al., 2014).

Garlic scape powder at 400, 800, and 1200 mg/kg bwt reduced body weight gain in hyperlipidemic rats by decreasing food intake. (Mofrad et al., 2019). Previously, garlic was an active ingredient that may reduce weight gain by decreasing glucose levels and promoting insulin sensitivity due to GO, DATS, and alliin (Sanie‐Jahromi et al., 2023).

The current study confirms a decrease and sustained serum insulin levels in different doses compared to the hyperlipidemia‐induced group (Zhai et al., 2018). Meanwhile, the hyperlipidemia‐induced group experienced an increase in serum insulin due to decreased insulin sensitivity and increased insulin resistance (Mukhtar et al., 2019).

The groups treated with garlic scape powder showed a dose‐dependent reduction in serum TC, TG, and LDL. After 12 weeks, the highest reduction in total cholesterol (88.1 ± 0.85 mg/dL) was seen with the highest dose of 1200 ppm/kg bwt, compared to hypercholesteremic rats (122.4 ± 1.48 mg/dL). TG levels (88.7 ± 1.64 mg/dL) and LDL levels (12.1 ± 1.81 mg/dL) were also lower compared to the hyperlipidemia group (132.6 ± 2.08 mg/dL) and the control group (39.4 ± 0.87 mg/dL), with the highest dose of 1200 mg/kg bwt.

The STD group received fenofibrate (20 mg/kg bwt), which specifically targets TG by activating transcriptional factor peroxisome proliferator‐activated receptors, encouraging cellular absorption of fatty acids. (Nakao et al., 2015). It is less likely for very low‐density lipoprotein (VLDL) to be made because of b‐oxidation pathways that break it down, conversion to acyl‐CoA derivatives, and less production of fatty acids and TG (Staels et al., 1998; Nimptsch et al., 2019). In conclusion, increased catabolism of TG‐rich particles and reduced production of VLDL mediate the hypotriglyceridemic action of fibrates, while changes in HDL apolipoprotein expression mediate their impact on HDL metabolism (Ahmed et al., 2022).

Garlic may lower LDL‐C levels by decreasing hepatic cholesterol 7α‐hydroxylase, HMG‐CoA reductase, pentose‐phosphate pathway activities, microsomal TG transfer protein, cholesteryl ester transfer protein activity, and enhancing bile acid excretion (Nakhaei et al., 2023; Onyeike et al., 2012).

At the fourth and eighth weeks, apoprotein E significantly decreased in PC compared with other groups. At the 12th week, apoprotein E levels increased in all treatment groups. At the highest dose of G3 (6.08 ± 0.010) compared to the group with hyperlipidemia (3.22 ± 0.03) and the highest dose of 1200 mg/kg bwt of garlic scape powder compared to other treatment groups and the STD group (Herz & Bock, 2002). Transporting lipids between the body's numerous cells and tissues is one of apoprotein E main jobs. Transporting lipids between the body's numerous cells and tissues is one of apoprotein E main jobs (Wu et al., 2023). In addition to playing a role in the homeostatic regulation of tissue and plasma lipid content, apoprotein E is a critical regulator of plasma lipid levels. Apoprotein E interacts with a high affinity for cell‐surface lipoprotein receptors, which helps achieve this. Mahley et al., (2007)  say that apoprotein E makes it easier for lipoproteins and lipid complexes that contain apoprotein E to connect to the LDL receptor, the LRP, the VLDL receptor, gp330, and the apoprotein E receptor 2.

A study on the distribution of apoprotein E in different plasma lipoproteins found that apoE4 prefers to connect with large, TG‐rich VLDL particles, while apoprotein E_3_ and apoprotein E_2_ prefer to connect with small, phospholipid‐rich HDL particles (Mahley, 2016).

Compared to the hyperlipidemia group, garlic scapes showed an increase in serum albumin at various dose levels. Garlic scapes' high content of free amino acids, ranging from 1121.7 to 3106, increased the serum albumin level at different dose levels. In this study, 1 mg/100 g of fresh weight (Ahmed & Wang, 2021). In the fourth and eighth weeks, albumin decreased in the hyperlipidemia group compared to other groups (Weigle et al., 2005). The albumin level went up in treatment group G3 (4.78 ± 0.04) at 12 weeks, with the highest dose of 1200 mg/kg bwt. This was different from STD (4.04 ± 0.09) and PC (2.95 ± 0.05) at 12 weeks (Ulla et al., 2017).

ALT and AST levels went up in the hyperlipidemia group, while garlic scape levels went down in different dose‐treated groups. Higher levels of the AST, ALT, and ALP enzymes in the plasma of rats that ate a lot of fat show that oxidative damage to tissues also hurts hepatocytes. These enzymes are regarded as indicators of liver dysfunction (Pradere et al, 2013). Hepatocyte injury typically transfers these enzymes to the plasma. Rats fed an HFD had higher plasma levels of TG and cholesterol, which led to the development of lipotoxicity and lipid buildup in the liver (Oforibika & Oforibika, 2020). The accumulation of fat in the liver leads to steatosis, also known as nonalcoholic fatty liver disease (NAFLD). Researchers have proposed a “two‐hit” theory to explain how an HFD triggers the development of NAFLD (Abdelrahman et al., 2023).

Oxidative injury and liver inflammation can also lead to extracellular matrix deposition and fibrosis in the liver (Haga et al., 2015). Rats on an HFD have shown hepatic fibrosis, but supplementing with foods high in antioxidants prevents it (Nadig et al., 2023).

Free radicles play important roles in activating hepatic stellate cells (HSCs) and making extracellular matrix, mostly collagen‐type proteins. Local immune cells, primarily Kupffer cells, stimulate HSCs to increase the production of extracellular matrix proteins. Kupffer cells produce TGF‐ and platelet‐derived growth factor, profibrotic cytokines that stimulate HSCs (Hossen, 2022).

The examination of the liver tissues in the NC under normal conditions revealed normal hepatocyte architecture and the usual size of the portal vein, as illustrated in Figure A. In the PC group, on the other hand (PC and G1), histological analysis showed that hepatocytes were destroyed, the portal vein was deformed, and cell contents were lost. In the STD (400 mg/kg bwt) group, rats' livers showed average portal vein size and distortion of the normal architecture. In G2 and G3 (800 mg/kg bwt and 1200 mg/kg bwt), treated groups restored normal histological structure, showing normal hepatic parenchyma.

**FIGURE 13:**
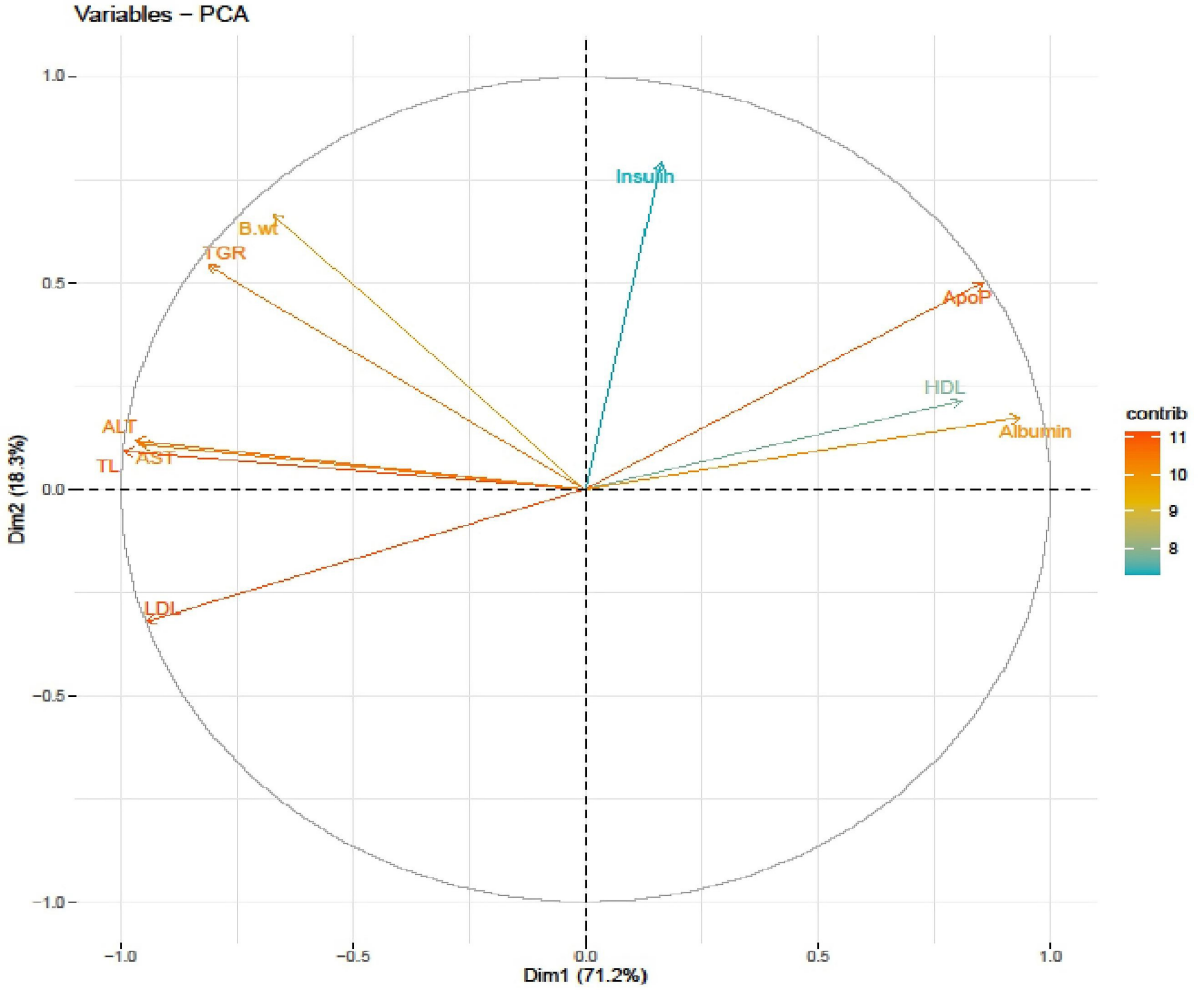
A principal component analysis shows a correlation among various attributes of the study. ALT, alanine aminotransferase; AST, aspartate aminotransferase; Bwt, body weight; LDL, low‐density lipoprotein; TGR, triglycerides; TL, total cholesterol.

Principle component analysis (Figure 13) showed the contribution of Dim1 (71.2%). The Dim2 part (18.3%) had a positive relationship with serum insulin, TG, TC, AST, ALT, LDL, and body weight (Bwt). On the other hand, it had a negative relationship with these same substances. In conclusion, garlic scape powder decreases cholesterol, TGR, LDL, and liver enzymes, which in turn increases insulin levels to enhance glucose utility, lowers lipolysis, and increases protein synthesis, thereby reducing the disease burden.

## 5 CONCLUSIONS

In the current long‐term study, rats were administered garlic scape powder at various doses for 12 weeks. The highest dose of garlic scape powder, 1200 mg/kg bwt, provided lipid‐lowering benefits due to increased cholesterol consumption by increasing apoprotein E levels and a better safety profile in hepatotoxicity. The garlic scapes positively affect the apoprotein E level, which increases cholesterol metabolism and reduces cardiovascular disease risk. Apoprotein E is associated with faster cholesterol clearance and a decreased risk of atherosclerotic cardiovascular disease. New treatments that target apoprotein E or its receptors have lowered LDL‐C levels and reduced the risk of cardiovascular disease. The higher dosage of garlic scapes produced a significantly higher apoprotein E, an essential contribution to cholesterol reduction. Regularly using garlic scapes is crucial for preventing and treating cardiovascular diseases.

## AUTHOR CONTRIBUTIONS

**Nizwa Itrat:** Conceptualization (equal); formal analysis (equal); writing – original draft (equal). **Mahr un Nisa:** Conceptualization (equal); methodology (equal); supervision (equal). **Fahad Al‐Asmari:** Funding acquisition (equal); software (equal); validation (equal); writing – review and editing (equal). **Mohamed Fawzy Ramadan:** Funding acquisition (equal); validation (equal); visualization (equal). **Eliasse Zongo:** Data curation (equal); funding acquisition (equal).

## CONFLICT OF INTEREST STATEMENT

The authors declare no conflict of interest.

## Data Availability

Not applicable.
